# A novel inflammatory bowel disease registry powered by artificial intelligence and natural language processing

**DOI:** 10.1371/journal.pdig.0001603

**Published:** 2026-08-27

**Authors:** Jeremy Liu Chen Kiow, Cristian Massaro, Efrain Cruz Jimenez, Itay Kalisky, Genelle Lunken, Yvette Leung, Brian Bressler, Greg Rosenfeld

**Affiliations:** 1 Department of Medicine, University of British Columbia, Vancouver, Canada; 2 Division of Gastroenterology, St. Paul’s Hospital, Vancouver, Canada; 3 IBD Centre of BC, Vancouver, Canada; 4 Division of Gastroenterology, Montreal University Hospital Centre (CHUM), Montreal, Canada; 5 Department of Pediatrics, University of British Columbia, Vancouver, Canada; 6 BC Children’s Hospital Research Institute, Vancouver, Canada; CHOP: The Children’s Hospital of Philadelphia, UNITED STATES OF AMERICA

## Abstract

Despite substantial recent progress in inflammatory bowel disease (IBD) therapies, there remains a large proportion of patients who are non-responders or develop significant adverse events. Accurate data registries may assist clinicians and researchers to gain insights into IBD and provide opportunities to improve overall patient care. Most data registries are limited by the amount of time and resources needed to collect and record patient-level data. We describe and validate a novel IBD repository (IBD Data Lake), leveraging machine learning and natural language processing (NLP) techniques, as useful tools to curate and retrieve pertinent, real-time clinical data in the IBD population. Structured and unstructured data were extracted from patients’ electronic medical record and were transferred to a secure cloud infrastructure and curated into a searchable database. A customized user interface was created, and an advanced NLP service (Comprehend Medical) was employed to extract clinical information from unstructured data from medical documents. Chart review served as the gold standard for validation. Between July 1, 2018 and July 31, 2023, 208 (104 IBD and 104 matched non-IBD) patients were retrieved. The IBD Data Lake’s performance metrics for identifying IBD patients were as follows: sensitivity 98.1%, specificity 97.1%, and accuracy 97.6%. The machine learning/NLP components demonstrated high performance in analyzing key IBD characteristics: for distinction of ulcerative colitis or Crohn’s disease, sensitivity was 100% and specificity 98.2%; for smoking status, sensitivity was 100% and specificity 96.9%; and for extraintestinal manifestations, sensitivity was 92% and specificity 100%. A novel IBD Data Lake that integrates machine learning and NLP techniques has been established and validated. IBD patients were identified with high accuracy and the machine learning/NLP components enable comprehensive and timely data extraction. This will ultimately lay the groundwork for recruitment of specific IBD cohorts of interest to address the gaps that remain in our knowledge.

## Author summary

Inflammatory bowel disease is a lifelong condition that affects millions of people worldwide. Although many new treatments have become available, many patients still do not respond well to therapy or experience side effects. Better clinical data are needed to understand why patients respond differently and to develop more personalized approaches to care. However, creating and maintaining high-quality disease registries is often slow, costly, and requires extensive manual review of medical records. In this study, we developed and validated a novel inflammatory bowel disease repository that uses artificial intelligence to enable faster and more comprehensive retrieval and organization of information from both structured records and physicians’ clinical notes. We found that our system accurately identified patients with inflammatory bowel disease and reliably extracted important clinical characteristics from their medical records. By reducing the need for manual data collection while providing timely and comprehensive information, our repository has the potential to accelerate clinical research and improve patient care. It also creates a foundation for identifying and following specific groups of patients, supporting future studies in personalized medicine, treatment outcomes, and disease management. This approach could help answer important clinical questions more efficiently in the field of gastroenterology and inflammatory bowel disease.

## Introduction

Inflammatory bowel disease (IBD), the two main subtypes being ulcerative colitis (UC) and Crohn’s disease (CD), is a group of chronic immune-mediated disorders that cause relapsing and remitting inflammation of the gastrointestinal tract [1]. IBD is a lifelong incurable condition characterized by debilitating symptoms of abdominal pain, diarrhea, rectal bleeding, weight loss, and fatigue. Patients often experience physical, social, and psychological distress, which can significantly impact their quality of life [2]. The exact etiopathogenesis of IBD is unknown, but it is thought to be multifactorial comprising environmental factors such as dietary habits, genetic predispositions, and an imbalance in (dysbiosis) as well as a dysregulated immune response to commensal gut microbiota [3]. The prevalence and incidence of IBD in Canada, which are among the highest in the world, have been increasing throughout the years and it has been estimated that by 2030, approximately 1% of the population will be living with IBD [4]. The troubling upward trend of IBD cases worldwide has made it a significant public health concern, underscoring the urgency for novel approaches in understanding and managing this complex condition [5].

Despite substantial progress in IBD research expanding our therapeutic armamentarium over the last decade, there remains a large proportion of patients who are primary (30%) and secondary non-responders (50%) or develop significant adverse events that require discontinuation of therapy [6,7]. One of the suggested approaches to overcome this therapeutic ceiling is through personalized and precision medicine strategies, which involve exploring and utilizing prognostic and predictive biomarkers to better stratify patients based on disease phenotype, risk of progression, and treatment response, in the hopes of improving overall clinical care in IBD and address the unique characteristics of each patient’s disease [8]. In this context, with the era of big data and the recent advancements that have been made in artificial intelligence (AI), there have been an abundancy of new clinical and research opportunities for expediting the development of advanced precision medicine applications that are both clinically relevant and useful in the field of IBD and gastroenterology [9]. These technologies hold the potential to revolutionize the field of IBD research and therapy by unlocking intricate patterns within vast datasets. The potential applications of AI in IBD include insight provision into the underlying molecular mechanisms to better understand the pathogenesis, increase in diagnosis accuracy and grading of severity, prediction of treatment response or failure, prognostication, and reduction of variation of care delivery and outcomes among different healthcare professionals and institutions [10].

Although machine learning and AI have allowed medical researchers to study disease-related trends, patterns, and complex associations to further our knowledge in IBD, the complexity of big data and the accompanying confounding factors make it very important for us to have comprehensive, high-quality data. Most sources of data in the field of IBD come from clinical trial registries, study cohorts, administrative databases, patient support program databases, and electronic health records [11]. Indeed, in order for these data to be of high quality, they should be intrinsically good (objective, accurate, and reputable), align with the relevant context (timely and complete), be presented in a clear manner (interpretable and consistent), and be readily accessible to those who use it [12]. Thus, creating biobanks and reliable data infrastructures, including databases (systems that store structured data in an organized and queryable format), data lakes (centralized repositories that store large volumes of raw structured and unstructured data), data warehouses (curated repositories that store cleaned, processed data prepared for analysis), and clinical registries (structured datasets containing key clinical variables for a specific disease), with the aim of generating data of the highest quality can help clinicians and health care providers develop useful tools and algorithms that ultimately support the goal of personalized medicine. Although administrative health databases have been very helpful in designing population-based studies to understand real-world experiences, their limitations lie in the potential selection biases and confounding factors, and lack of specific patient level clinical data [17–19]. Furthermore, patient registries provide important insights into disease progression, treatment responses, and patient outcomes, all of which are essential for enhancing overall care in chronic diseases. However, current registries often face practical challenges due to their reliance on dedicated data entry personnel, leading to human error and inconsistencies that possibly compromise data accuracy and reliability. Additionally, the necessity for manual data input results in high costs and resource utilization.

To address the issues related to the practical barriers faced by existing registries and the critical need in precision medicine, the objective of our study was to develop and validate a novel IBD Data Lake, leveraging machine learning and natural language processing (NLP) techniques, as useful tools to curate and retrieve relevant, real-time clinical data in the IBD patient population. We also aimed to establish automated data collection from electronic medical records as a high-quality data source for both research and clinical care. This innovative resource strives to enhance our opportunities for clinical research and optimal care in the field of IBD, but also paves the way for the development of more precise and effective personalized medicine applications. This initiative will establish the foundation for identifying, monitoring, and studying specific IBD cohorts of interest, with the goal of addressing existing knowledge gaps. Here, we present the creation, development, and validation of our IBD Data Lake and shed light on its potential to drive groundbreaking advancements in the field of IBD and gastroenterology.

## Materials and methods

### Ethics statement

Informed consent was obtained from patients by the IBDCBC gastroenterologists to include their data in our IBD Data Lake. This validation study was approved by the University of British Columbia Providence Health Care Research Ethics Board.

### Creation and description of the IBD Data Lake

The IBD Data Lake was created through the collaboration of gastroenterologists, IBD specialists, researchers, data strategists, data scientists, and software engineers with experience in NLP and machine learning at the IBD Centre of British Columbia (IBDCBC). A backup of the electronic health record of the IBDCBC, which supports a large and specialized tertiary IBD practice, was generated, spanning five years up to July 31, 2023. This involved securely transferring and storing all relevant structured (diagnostic codes, billing codes, demographics, and laboratory values) and unstructured (clinical notes and medical documents) data on the Amazon Web Services (AWS) cloud infrastructure to ensure reliability, scalability, and data durability. The data encompassed all available medical records generated during routine IBD care, including outpatient consultation and progress notes, colonoscopy reports, histopathology reports, and imaging reports. Although all note types were used for feature extraction, the most recent consultation or progress note per patient was prioritized, as it was considered the most clinically reliable reflection of current disease status. All AWS services were provided through the Provincial Health Services Authority (PHSA), a publicly funded health authority in the province of British Columbia that manages and oversees safety aspects of this Canadian-based cloud environment.

To facilitate the extraction of unstructured information within the cloud-based repository, we first implemented Amazon Textract, an Optical Character Recognition (OCR) service used to obtain computer-readable data. More specifically, it facilitated the recognition and extraction of typed and handwritten text or data from scanned documents, including PDF files. Textract systematically processed these documents, converting scanned images or files into machine-readable text, enabling further processing of the information [13].

Subsequently, Amazon Comprehend Medical (ACM), an NLP service provided by AWS, was employed to extract pertinent medical information from the processed unstructured text and data from documents. This step involved utilizing advanced machine learning algorithms, commercially pre-built and pre-trained, to identify and comprehend various healthcare-related details such as diagnoses, medications, clinical reports, and medical procedures [14]. The data were then structured in the data lake to facilitate clinical use.

**Fig 1:**
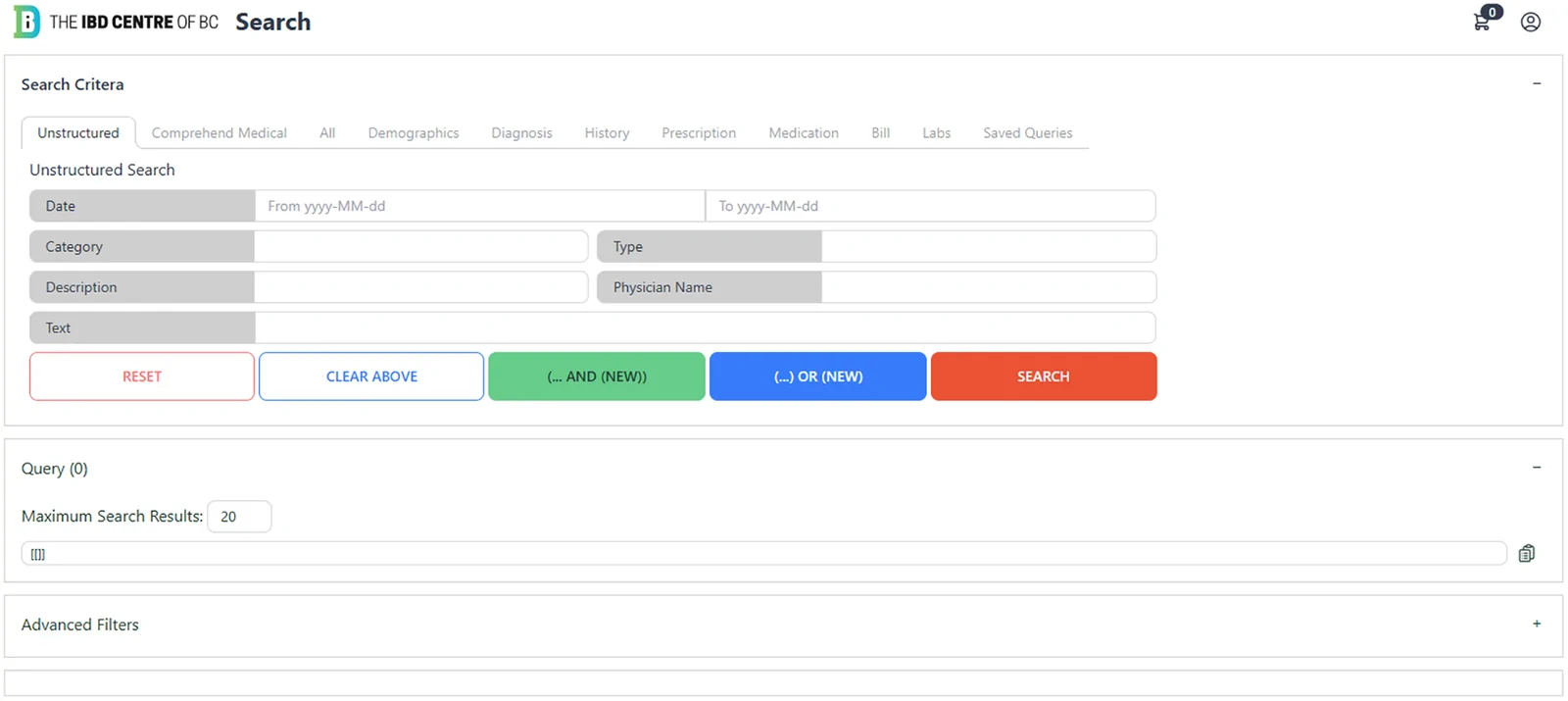
The IBD Data Lake user interface.

**Fig 2:**
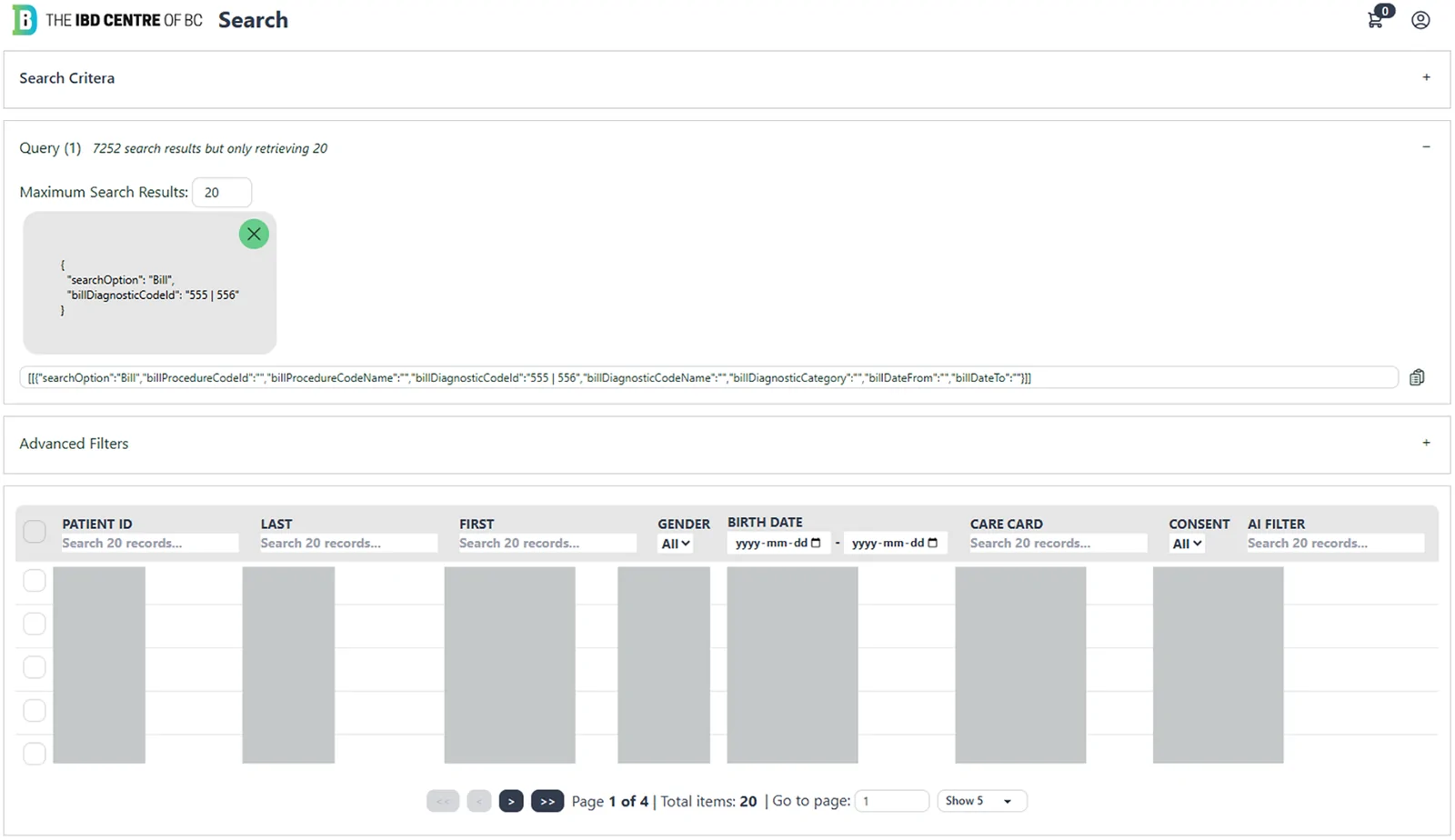
The IBD Data Lake user interface illustrating a sample search query.

To enhance user interaction and accessibility, a user-friendly interface was developed, resembling the familiar design of popular search engines, as shown in Fig 1 and Fig 2. This was achieved through the integration of Amazon Cognito for flexible user authentication and OpenSearch, a search and analytics engine, for efficient data retrieval and visualization when processing large volumes of data. In our case, the dataset managed was over 400 GB in size and therefore, using standard tools would make this task infeasible or unrealistic. The user interface aimed to provide a unified experience similar to widely used platforms and research search engines (e.g., PubMed and Embase), simplifying the exploration and analysis of the extracted medical data [15,16]. Physicians at the IBD Centre collaborated with the development team and engaged in iterative testing to ensure clinical functionality and validate the accuracy and consistency of automated data retrieval.

Throughout the entire implementation process, utmost attention was given to data security, patient consent, and compliance with ethics and health regulations. With all the services provided by AWS, encryption protocols were employed to preserve sensitive patient information during transmission and storage. The services were in alignment with best practices in data protection, privacy, and security. Moreover, continuous monitoring and improvement mechanisms were established to enhance system performance, adapt to evolving healthcare data formats, and address information security risks. Regular updates and refinements were implemented to optimize the system’s overall efficiency and maintain its relevant applications.

By combining an extensive cloud storage backup, OCR technology, NLP capabilities with machine learning, and a convenient user experience, our IBD Data Lake provides a comprehensive method for extracting, analyzing, and visualizing pertinent clinical information from electronic health records in a timely manner. This approach was implemented to simplify data management and contribute to the efficiency and accuracy of data extraction.

### Study design and inclusion criteria

This was a validation study of our IBD Data Lake. Chart review using our patient electronic health record was used as our gold standard to validate the diagnosis of IBD and other relevant clinical information retrieved from the IBD Data Lake. Two authors (JLCK, CM) independently performed the validation tasks, including data extraction and comparison. Adult patients, 18 years or older, followed at the IBDCBC between July 1, 2018 and July 31, 2023 and diagnosed IBD based on standard clinical, radiological, endoscopic, and histologic criteria were included in the study. In order to measure the diagnostic accuracy, including the sensitivity and specificity, of the Data Lake in identifying patients with IBD, we included a matched group of non-IBD patients who had a colonoscopy for similar gastrointestinal symptoms and/or for screening purposes following a positive fecal immunochemical test (FIT). The lists of patients for both groups were generated by the IBD Data Lake on March 22, 2024.

### Data collection

Each subject was assigned a unique study identification number. The participant details were first extracted from our IBD Data Lake and from our electronic health record for comparison and validation. Demographic features (age and gender) and all necessary clinical (clinical notes reporting gastrointestinal symptoms), biochemical (CRP and fecal calprotectin), endoscopic (colonoscopy and gastroscopy), radiologic (CT scan and MRI), and histologic (gastrointestinal biopsies) data to confirm the diagnosis of IBD were collected. In order to better describe our chosen patient population, IBD disease characteristics (Montreal classification) were also extracted, as well as the presence of extraintestinal manifestations (EIM) and smoking status when available.

### Study outcomes

The primary outcome of the study was to evaluate the diagnostic accuracy (sensitivity, specificity, positive predictive value [PPV], and negative predictive value [NPV]) of the IBD Data Lake in identifying patients diagnosed with IBD. Secondary outcomes included assessing the diagnostic accuracy and performance of ACM in extracting relevant clinical information from the IBD cohort, such as IBD subtype, smoking status, and EIM.

### Validation process

To identify and gather all British Columbia residents followed at the IBDCBC with a diagnosis of IBD in our Data Lake, the International Classification of Diseases, Tenth Revision (ICD-10), codes 555 and 556 were used for CD and UC, respectively. The majority of British Columbia physicians practice on a fee-for-service basis, including our IBD specialists at the IBDCBC, and need to submit a claim that includes the patient’s diagnosis according to the appropriate ICD-10 codes. A random sample of IBD patients using the ICD-10 codes 555 and 556 was chosen from the generated list.

Patients without a corresponding ICD-10 code for IBD, who had a colonoscopy for gastrointestinal symptoms and/or for screening purposes (positive FIT test), were identified based on the following billing codes: 10731 (diagnostic colonoscopy), 3373 (diagnostic colonoscopy with biopsies), and 3374 (diagnostic colonoscopy and polypectomy).

A structured chart review was performed to categorize patients into the following categories: 1) diagnosis of CD; 2) diagnosis of UC; 3) no diagnosis of IBD. The diagnosis of IBD, including CD and UC, was based on standard clinical, endoscopic, radiologic, and histologic criteria. The comprehensive chart review was used as a gold standard for validation. This ultimately allowed us to calculate the sensitivity, specificity, PPV, and NPV of our IBD Data Lake in identifying all patients with IBD.

The NLP and OCR components of the IBD Data Lake were also explored throughout the process and their performance (shown as confidence score) in accurately identifying clinical information from our cohort of IBD patients was compared to the chart review. ACM provided a confidence score which indicated the level of confidence in the accuracy of the detected entities or attributes. Following consultation with AWS, confidence score thresholds for ACM were defined according to empirical performance characteristics and widely accepted model evaluation methodologies. A high confidence score was defined by a confidence score ≥ 0.8, indicating that the ACM service was highly confident of the accuracy of the identified entity and/or attribute. A confidence score between 0.6 and 0.8 was defined as a moderate confidence score, suggesting that the identified information was likely correct, but with some uncertainty. A low confidence score, < 0.6, indicated that the identified entity may not be accurate, and that the information should be reviewed carefully.

Occasional instances of missing or incomplete outputs were encountered during processing of unstructured data with ACM. These cases represented a small proportion of the dataset. When ACM did not return a result, we applied a conservative and systematic approach by treating the absence of an output as an unfavorable (negative) finding. This rule was applied consistently to avoid inflating positive results and was considered appropriate to maintain analytical rigor and prevent bias introduced by data gaps.

### Statistical analysis and sample size

The sensitivity, specificity, PPV, NPV, and accuracy of our IBD Data Lake were calculated. Assuming a sensitivity of at least 90%, a total sample size of 139 patients, split equally between IBD and non-IBD cohorts, was necessary to achieve a margin of error of 5% at a 95% confidence level. This sample size ensured that the resulting confidence interval, for a sensitivity of 90%, would range from 85% to 95%. Similar assumptions and calculations were done for specificity. Although a larger sample size would lead to greater precision, a sensitivity and specificity of at least 90% was deemed adequate based on previous evidence from IBD health administrative data.

Wilson score confidence intervals were calculated for all performance metrics to provide more robust interval estimates, particularly given the small number of misclassified cases. In addition, F1 scores were calculated for each machine learning/NLP-derived classification task to complement sensitivity and specificity and to better characterize model performance in the presence of class imbalance.

## Results

### Study demographics

**Fig 3:**
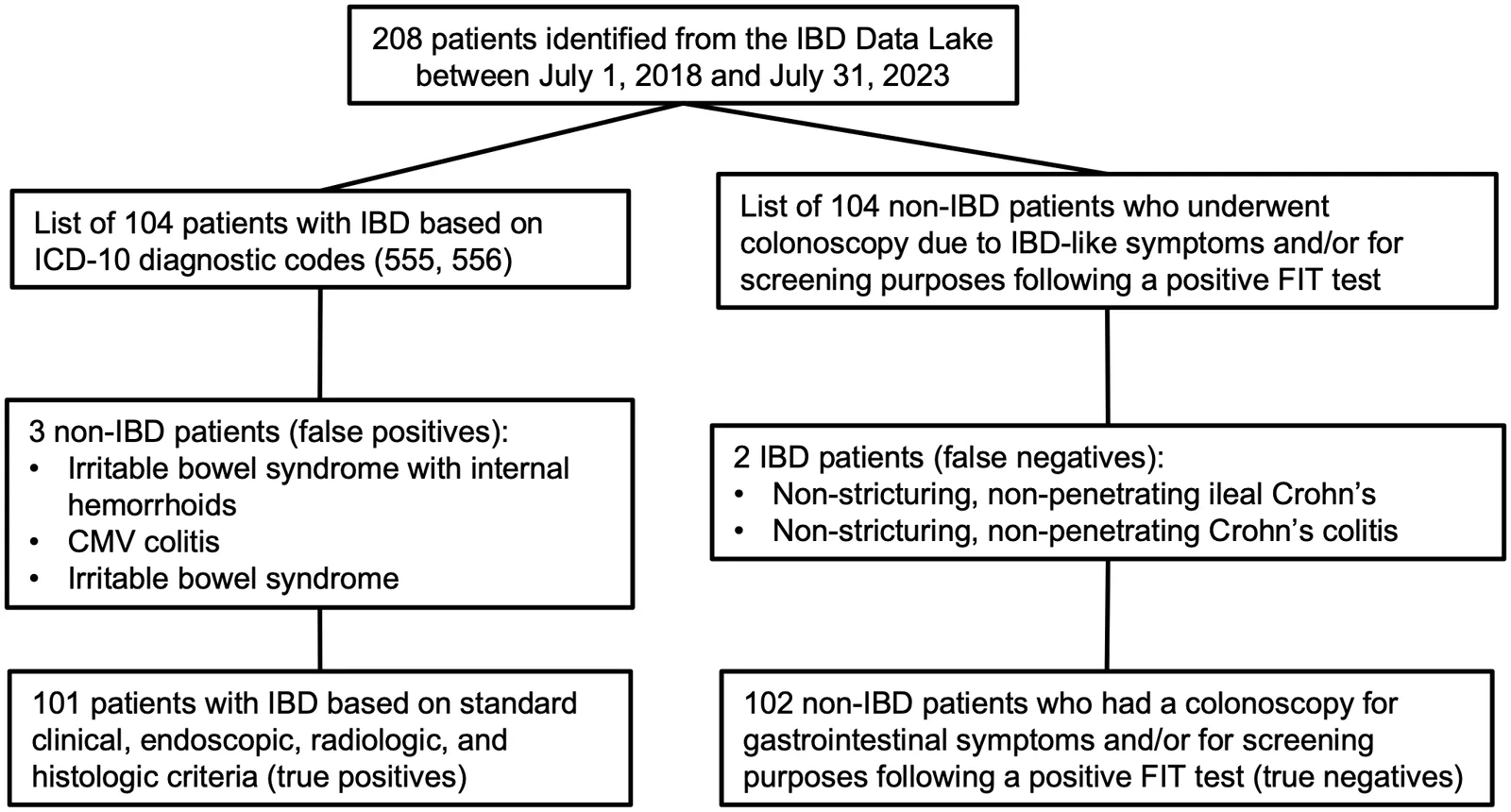
Groups of patients generated by the IBD Data Lake for study inclusion.

Between July 1, 2018 and July 31, 2023, a total of 208 patients were identified from the IBD Data Lake who satisfied criteria for inclusion in this validation study. Patient selection for study inclusion is outlined in **Fig 3**. Two groups of patients were generated: the IBD cohort included 104 patients, matched with 104 non-IBD patients who underwent colonoscopy either due to IBD-like symptoms or for screening purposes following a positive FIT test. After a thorough chart review, 101 out of 104 patients met the diagnostic criteria for IBD in the IBD cohort. In contrast, the non-IBD cohort comprised 102 patients, with 2 cases of IBD that were not initially identified. Baseline characteristics are summarized in **Table 1**. Of the IBD cohort, 58 patients had CD (57.4%) and 43 patients had UC (42.6%). The majority were non-smokers (94%), males (53.5%), and a quarter had EIM of their IBD. In the non-IBD cohort, patients were older and the majority, just over three quarters, had a colonoscopy following a positive FIT test. Approximately 40% had gastrointestinal symptoms warranting further investigations. Colonoscopies in the non-IBD cohort were normal in 46% of cases and half of the patients had colorectal polyps. Most patients of the non-IBD cohort were non-smokers as well (88%), but with a slight female predominance (55%).

**Table 1:** Baseline characteristics of inflammatory bowel disease and non-inflammatory bowel disease cohorts.

	IBD (n = 104)	Non-IBD (n = 104)
Diagnosis discrepancies, n (%)	3 (2.9)	2 (1.9)
IBD diagnosis confirmed (true positives), n (%)	**101 (97.1)**	–
Non-IBD diagnosis confirmed (true negatives), n (%)	–	**102 (98.1)**
Male gender, n (%)	54 (53.5)	46 (45.1)
Age at study start (years ± SD)	39.5 ± 14.2	58.0 ± 12.2
Age at IBD diagnosis (years ± SD)	26 ± 13.4	–
CD, n (%)	58 (57.4)	–
UC, n (%)	43 (42.6)	–
Smoking, n (%)	6 (5.9)	12 (11.8)
Previous smoker	6 (5.9)	6 (5.9)
Non-smoker	88 (87.1)	82 (80.4)
CD location, n (%)		
Ileal	19 (32.8)	–
Colonic	13 (22.4)	–
Ileocolonic	27 (46.6)	–
Isolated upper disease	1 (1.7)	–
Isolated perianal disease	1 (1.7)	–
CD Behaviour, n (%)		
Stricturing	17 (29.3)	–
Penetrating	11 (19.0)	–
Perianal fistulizing disease	17 (29.3)	–
Non-stricturing, non-penetrating	28 (48.3)	–
UC extent, n (%)		
Proctitis	15 (34.9)	–
Left-sided	12 (27.9)	–
Pancolitis	16 (37.2)	–
Extraintestinal manifestations, n (%)	27 (26.7)	–
Skin	9 (33.3)	–
Joint	19 (70.4)	–
Eye	2 (7.4)	–
PSC	1 (3.7)	–
Oral	3 (11.1)	–
None	73 (72.3)	–
Colonoscopy indication in non-IBD cohort, n (%)		
GI Symptoms	–	40 (39.2)
Screening purposes	–	78 (76.5)
Findings on colonoscopy in non-IBD cohort, n (%)		
Normal	–	47 (46.1)
Polyps	–	51 (50.0)
Colorectal cancer	–	1 (1.0)
Other	–	3 (2.9)^1^
Diagnosis in non-IBD cohort, n (%)		
IBS/functional	–	11 (10.8)
Polyps	–	46 (45.1)
Hemorrhoidal bleed	–	11 (10.8)
Colorectal cancer	–	1 (1.0)
Other	–	3 (2.9)^2^
No diagnosis confirmed or idiopathic		30 (29.4)

IBD – Inflammatory bowel disease

CD – Crohn’s disease

UC – Ulcerative colitis

PSC – Primary sclerosing cholangitis

^1^Aphthous ulcers x 2, pneumatosis coli

^2^Anal fissure, drug-induced diarrhea (Metformin), medication adverse effect (Aspirin), idiopathic

### Validation of IBD study population

In the IBD cohort, 3 patients were misclassified as having IBD (false positives). Therefore, 101 patients actually had IBD (true positives). Conversely, in the non-IBD cohort, 2 patients were misclassified as not having IBD (false negatives). Hence, 102 patients did not have IBD (true negatives) but had a colonoscopy for gastrointestinal symptoms or for screening purposes following a positive FIT test (**Fig 3**).

The sensitivity, specificity, PPV, NPV, and accuracy our of IBD Data Lake in identifying patients with IBD, based on the corresponding ICD-10 codes, were 98.1% [95% CI, 97.0-98.8%], 97.1% [95% CI, 95.9-98.0%], 97.1% [95% CI, 95.9-98.0], 98.1% [95% CI, 97.0-98.8%], and 97.6% [95% CI, 96.5-98.4%], respectively (**Table 2**).

**Table 2:** Validation of the Inflammatory Bowel Disease Data Lake. Sensitivity, specificity, positive predictive value, negative predictive value, and diagnostic accuracy with 95% CI of the Data Lake in identifying inflammatory bowel disease diagnosis and clinical characteristics.

Variable	Sensitivity (95% CI)	Specificity (95% CI)	PPV (95% CI)	NPV (95% CI)	Accuracy (95% CI)	Youden’s Index	F1 Score (%)
**IBD diagnosis**	98.1% (97.0-98.8)	97.1% (95.9-98.0)	97.1% (95.9-98.0)	98.1% (97.0-98.8)	97.6% (96.5-98.4)	0.95	97.6
**IBD subtype** **(UC versus CD)**	100% (90.1-100)	98.2% (90.1-100)	97.5% (84.8-99.6)	100% (93.3-100)	98.9% (94.2-100)	0.98	97.9
**Smoking**	100% (39.8-100)	96.9% (89.3-99.6)	66.7% (33.8-88.7)	100% (94.3-100)	97.1% (89.9-99.7)	0.97	57.1
**EIM**	92.0% (74.0-99.0)	100% (92.1-100)	100% (85.2-100)	95.7% (85.6-98.8)	97.1% (90.1-99.7)	0.92	88.4

IBD – Inflammatory bowel disease

CI – Confidence interval

CD – Crohn’s disease

UC – Ulcerative colitis

EIM – Extraintestinal manifestations

### Validation of machine learning and NLP components of the IBD Data Lake

Machine learning and NLP results of the IBD Data Lake were reviewed in the 101 IBD patients from the IBD cohort, specifically looking at relevant unstructured clinical data including IBD subtype, smoking, and EIM. Using ACM results for each of these variables, the sensitivity, specificity, PPV, NPV, and accuracy are described in **Table 2**.

The IBD Data Lake’s ACM correctly identified over 90% of IBD patients, including their IBD subtype (UC versus CD). The average confidence score, the level of confidence in the accuracy of the variable analyzed, was 96% for IBD diagnosis, 98% for CD, and 94% for UC. It also identified about two thirds of patients’ smoking status with an average confidence score over 90% for current smokers and non-smokers. For previous smokers, the average confidence score was lower, but still in the moderately to high confident range. The performance of the NLP in retrieving information for the Montreal classification varied, showing better results for the extent/location component of CD and UC, but slightly weaker performance for CD behaviour. More specifically, the ACM concordance, the number and percentage of patients correctly identified by ACM as compared to our chart review gold standard, ranged from 77-100% for CD location (lowest score for colonic CD), 32–88% for CD behaviour (lowest score for non-stricturing, non-penetrating CD), and 75–92% for UC extent (lowest score for pancolitis) (**Table 3**). However, the mean confidence score given by ACM when correctly identified was high: 90–99% for CD location, 81–95% for CD behaviour, and 90–92% for UC extent.

**Table 3:** Inflammatory Bowel Disease Data Lake’s natural language processing detailed diagnostic performance.

	IBD cohort, n (%)	NLP/ACM Concordance^1^, n (%)	NLP/ACM Discordance^2^, n (%)	Mean Confidence^3^ Score (%)
Total IBD cohort (true positives)	101 (100)	92 (91.1; 83.3-95.6)	1 (1.0; 0-6.20)	96.3
CD	58 (57.4)	54 (93.1; 82.5-97.8)	1 (1.72; 0-10.5)	97.8
UC	43 (42.6)	39 (90.7; 77.0-97.0)	0	94.3
Smoking	6 (5.94)	4 (66.7; 24.1-94.0)	2 (33.3; 5.99-75.9)	93
Previous smoker	6 (5.94)	3 (50.0; 14.0-86.1)	2 (33.3; 5.99-75.9)	81.7
Non-smoker	88 (87.1)	58 (65.9; 54.9-75.5)	1 (1.1; 0-7.00)	92.7
CD location				
Ileal	19 (32.8)	15 (78.9; 53.9-93.0)	–	95.6
Colonic	13 (22.4)	10 (76.9; 46.0-93.8)	–	96.5
Ileocolonic	27 (46.6)	24 (88.9; 69.7-97.1)	1 (3.7; 0-20.9)	93.9
Isolated upper disease	1 (1.7)	1 (100)	–	90.0
Isolated perianal disease	1 (1.7)	1 (100)	–	99.0
CD behaviour				
Stricturing	17 (29.3)	12 (70.6; 44.1-88.6)	–	86.3
Penetrating	11 (19.0)	8 (72.7; 39.3-92.7)	–	81.1
Perianal fistulizing disease	17 (29.3)	15 (88.2; 62.2-97.9)	1 (5.9; 0-30.8)	87.9
Non-stricturing, non-penetrating	28 (48.3)	9 (32.1; 16.6-52.4)	1 (3.6; 0-20.3)	94.7
UC extent				
Proctitis	15 (34.9)	13 (86.7; 58.4-97.7)	–	90.3
Left-sided	12 (27.9)	11 (91.7; 59.8-99.6)	–	90.3
Pancolitis	16 (37.2)	12 (75; 47.4-91.7)	–	92.0

For EIM, the overall ACM concordance was 85%, which means that the IBD Data Lake correctly identified 23 out of the 27 IBD patients who had EIM. The overall mean confidence score for EIM was very high at 96%. The IBD Data Lake was most effective at identifying skin lesions, joint manifestations, and primary sclerosing cholangitis among the different types of EIM (**Table 4**).

**Table 4:** Performance of the Inflammatory Bowel Disease Data Lake’s natural language processing component in detecting extraintestinal manifestations.

	IBD cohort, n (%)	NLP/ACM Concordance^1^, n (%)	NLP/ACM Discordance^2^, n (%)	Mean Confidence^3^ Score (%)
Extraintestinal Manifestations	27 (26.7)	23 (85.2; 65.4-95.2)	2 (7.4; 1.29-25.7)	95.7
Skin	9 (33.3)	8 (88.9; 50.7-99.4)	1 (11.1; 0-49.3)	99.6
Joint	19 (70.4)	16 (84.2; 59.5-95.8)	1 (5.3; 0-28.2)	94.7
Eye	2 (7.4)	1 (50.0; 2.67-97.3)	1 (50.0; 2.67-97.3)	99.0
PSC	1 (3.70)	1 (100)	–	86.0
Oral	3 (11.1)	2 (66.7; 12.6-98.2)	1 (33.3; 1.76-87.5)	98.0
None	73 (72.3)	44 (60.3; 48.2-71.4)	29 (39.7; 28.6-51.9)	82.3

IBD – Inflammatory bowel disease

NLP – Natural language processing

ACM – Amazon Comprehend Medical

CD – Crohn’s disease

PSC – Primary sclerosing cholangitis

^1^Represents the number and percentage of patients correctly identified by ACM

^2^Represents the number and percentage of patients incorrectly identified by ACM

^3^Represents the average confidence score given by ACM when the specific IBD characteristic was correctly identified

The corresponding Wilson confidence intervals for each performance metric were narrow, supporting the precision of the estimates. The F1 score further confirmed strong harmonic performance between precision and recall for the machine learning NLP classification tasks, particularly for IBD diagnosis, differentiation between UC and CD, and EIM detection.

## Discussion

Health administrative data represent a valuable resource for conducting robust, population-based studies assessing chronic diseases and conditions. Nevertheless, ensuring their accuracy through rigorous validation is crucial, as biases, particularly misclassification biases, pose a significant threat to the validity of studies relying on these datasets. Canadian IBD databases using comprehensive administrative health data have previously been validated and showed their utility in estimating the incidence and prevalence of these disorders and identifying potential clinically significant trends or patterns [17–19]. However, the extraction of meaningful or pertinent clinical information for each patient from electronic health records in a timely manner can be challenging, especially if they are in an unstructured format.

In addition to having structured clinical and demographic data readily accessible, our IBD Data Lake is unique and novel as it also has the NLP service, ACM, integrated facilitating the analytical processing of unstructured data for clinical or research purposes. These results demonstrate that our IBD Data Lake has successfully been validated in our IBD population with great diagnostic accuracy. More specifically, we can be confident in its ability to identify IBD patients, as its sensitivity, specificity, and accuracy of 98%, 97%, and 98%, respectively, prove it to be an accurate tool for gathering and studying a cohort of IBD patients. The high F1 scores further confirm that the model maintained balanced performance across precision and recall. Furthermore, the machine learning and NLP components within the Data Lake demonstrated high accuracy in identifying key IBD characteristics, including smoking status, IBD subtype, and EIM, from the unstructured clinical data. Notably, ACM excelled in detecting those variables with a sensitivity, specificity, and accuracy over 90% in all cases.

ACM, offered by AWS, is a specialized NLP service designed specifically for precise information extraction from unstructured medical texts or documents. This neural network-based tool processes diverse healthcare-related text sources, including clinical notes, reports, and prescriptions from patient health records, and utilizes advanced machine learning algorithms. By being pre-trained, it demonstrates the capability to identify, comprehend, and extract crucial details from unstructured data quickly and accurately. This includes diagnoses, medications, procedures, and other valuable medical information. The extracted health data are then automatically linked to specific medical ontologies (RXNorm and ICD-10-CM), or other standards extensively used [14]. In fact, ACM has been shown to be superior in performance, in terms of recall, precision, and F-score, when compared to some other entity extraction tools [20]. The application of such NLP using machine learning extends its value to healthcare providers, researchers, and organizations seeking to analyze and derive insights from substantial volumes of medical information and integrate big data analytics into clinical practice, in the hopes of contributing to more efficient and informed decision-making in the patient care.

When specifically examining the ACM confidence scores, which reflect the service’s confidence in the accuracy of the identified entities, the IBD Data Lake showed high confidence in identifying IBD patients, IBD subtype, CD location, UC extent, and EIM. Mean confidence scores for CD behavior and smoking status were slightly lower, but still within the moderate to high range. The concordance of the results (i.e., the accuracy in identifying patients by ACM) followed a similar pattern, with somewhat reduced scores for CD behavior and smoking history.

Smoking status was documented in a limited number of patients in this cohort, and performance metrics for this variable should be considered exploratory. Although binary detection sensitivity was high, the associated confidence interval was wide, reflecting the small sample size. Subcategory-level concordance was lower, primarily due to misclassification between current and previous smoking status. Validation in a larger cohort is needed before conclusions can be drawn regarding the model’s ability to distinguish smoking subcategories.

The variability observed in Montreal classification performance, particularly for Crohn’s disease behaviour, likely reflects a combination of factors. Disease behaviour and phenotype are often incompletely or inconsistently recorded in clinical notes when not central to the clinical encounter, limiting the available signal for extraction. This is compounded by the inherent constraints of ACM as a general-purpose pre-trained service that does not support custom model training or domain-specific optimization. In contrast, disease extent and location are more consistently documented in structured reports such as endoscopy and pathology, explaining the markedly higher performance for these parameters. Improving clinical documentation consistency and exploring customizable NLP platforms may further enhance performance for behaviour and phenotype classification.

While our study found a minimal occurrence of false positives and false negatives, it is important to acknowledge the potential impact of these errors, even if few. False positives, though rare, could lead to overestimating the system’s accuracy, while false negatives may result in missing critical findings. In a clinical context, such discrepancies could influence cohort selection or downstream analyses if not carefully monitored. Although these errors did not significantly affect the overall conclusions, they likely stem from inherent variability in clinical documentation or NLP interpretation of ambiguous text. Future system improvements should focus on reducing these occurrences further. Implementing more diverse data sources, refining algorithmic sensitivity, and exploring ensemble methods could help achieve this goal, ensuring even more reliable and accurate results in future iterations.

Interestingly, while compiling the machine learning and NLP data for analysis, ACM identified and corrected inaccuracies in the initial data collection from patient chart reviews. For instance, one case involved a patient who was misclassified as having CD when they truly had ulcerative proctitis. Another case involved a patient who was recorded as a cigarette smoker when, in fact, they only smoked marijuana. These discrepancies reflected errors in the original manual chart review, not autonomous changes made by the AI system, and the NLP outputs simply prompted a re-examination of the source records.

This main strength of our study lies in its novelty and its potential for further research and clinical use, supported by NLP and machine learning. While our IBD Data Lake includes structured data that can be queried easily, its uniqueness and greatest asset lie in its NLP functionality, enabling the retrieval and organized presentation of unstructured data – a capability not yet found in any other administrative health or IBD database. The Data Lake overcomes the challenge of human data entry and allows collection of data variables using routine clinical health records. Moreover, the research group involved in the development of the Data Lake comprised experts from various fields (IBD subspecialty gastroenterologists, data strategists/scientists, IBD dietitians, and translational researchers) and is affiliated with a Canadian IBD tertiary center that has extensive experience in managing one of the largest IBD cohorts in Canada. In conducting this validation study, we thoroughly reviewed existing IBD administrative data studies and performed power calculations to determine the appropriate sample size necessary for validation. Moreover, the IBD Data Lake may assist in conducting medical cohort analyses and selecting suitable patient groups for clinical trials while reducing costs. To the best of our knowledge, this is the first IBD Data Lake of its kind described, capable of retrieving important clinical information in a timely manner by leveraging machine learning and NLP. Looking ahead, the IBD Data Lake provides a strong foundation for integrating emerging large language model (LLM) technologies. While ACM reliably and efficiently extracts discrete clinical entities, LLMs offer the potential to interpret longitudinal narratives, generate structured outputs from unstructured text, and automatically summarize complex encounters. Incorporating LLM-driven workflows could further improve data completeness, reduce the need for manual abstraction, and enable real-time clinical decision support. In parallel, situating our platform alongside other emerging AI-enabled registries highlights a broader shift toward multimodal, automated health-data ecosystems. Within this landscape, LLM integration represents a natural and necessary evolution in expanding both the analytical depth and clinical utility of the IBD Data Lake.

There are several limitations to the present study. Not all parameters of the IBD Data Lake were validated. However, we believe that the chosen variables are representative of the data typically collected for clinical and research purposes. Validating all potential variables of the Data Lake would not be feasible and thus, only certain pertinent clinical characteristics were selected. Additionally, relying on ICD-10 codes as an initial method to identify potential IBD cases introduces inherent biases, as coding may be incomplete, inconsistent, or influenced by billing practices; this may lead to misclassification or missed cases and underscores the importance of subsequent validation through chart review. In our study, however, all patients included in the final sample were confirmed to have IBD through detailed chart review, indicating that any ICD-10 related misclassification had minimal impact on the cohort composition. While this study was conducted at a single tertiary IBD centre, which may limit the generalizability of the findings, our institution is among the largest in Canada and manages one of the country’s most extensive and diverse IBD cohorts. This enhances the representativeness of our results within a high-volume, complex care setting. The IBD Data Lake demonstrated strong performance metrics in this environment; however, referral patterns may introduce selection bias toward more severe or treatment-refractory cases typically seen in specialized centres. Furthermore, differences in electronic medical record systems, documentation practices, and healthcare infrastructure across institutions and jurisdictions may influence the performance and portability of similar NLP and machine learning models. Future work should therefore aim to validate the IBD Data Lake across multiple clinical sites and healthcare settings to ensure its reproducibility, scalability, and broader generalizability. Manual chart review of patients’ electronic health records was employed as the gold standard for comparison and validation, acknowledging the potential for human errors in data extraction that could bias the results. Interestingly, the IBD Data Lake was able to identify and correct some of those errors. Although this study focuses primarily on the validation and performance of the IBD Data Lake, the economic implications of implementing such a system warrant consideration. The initial setup and maintenance of a cloud-based infrastructure leveraging NLP and machine learning services require technical expertise and institutional investment. However, these costs may be offset by long-term efficiencies, including reduced reliance on manual data entry, decreased risk of transcription errors, and improved capacity for real-time data extraction and analysis. Over time, automation of clinical data curation could substantially reduce personnel hours required for registry upkeep compared with traditional methods. The IBD Data Lake’s requirement to create structured queries using operators like “AND” and “OR” may restrict users’ ability to query the registry in a more natural, human-like manner. Although the IBD Data Lake has NLP capabilities, only IBD patients have been processed by ACM. The goal was to have this tool exclusively for IBD patients; therefore, we cannot be certain of its accuracy in our non-IBD cohort. Moreover, not all clinical data and medical documents were processed by ACM due to the inherent constraints of our methodology. Specifically, we have chosen to process patients’ electronic health records covering approximately five years, up to July 31, 2023. At present, data are deposited into the IBD Data Lake on a monthly basis through a secure transfer from the electronic medical record vendor into our AWS environment, supported by automated scheduled transfers and standardized processing pipelines. It is possible that model performance may differ when applied in a real-time setting, where data are ingested continuously, and documentation patterns may vary across encounters. Going forward, our team will continuously process and update patients’ medical records within the IBD Data Lake in real time, ensuring that the data remain current. This will enable us to evaluate the stability, reproducibility, and performance of extraction prospectively within an operational, real-time environment. One of the objectives of this study was to establish automated data collection using NLP and machine learning to reduce reliance on manual data entry. Although our results focused primarily on validation performance, the successful integration of automated document processing and clinical NLP tools within the IBD Data Lake demonstrates that automated extraction of structured and unstructured clinical data is both feasible and accurate. Going forward, enhancing the automation and frequency of data ingestion will ensure continuously updated information and will enable more sophisticated data visualizations and comprehensive clinical analyses. As it stands, the information on medications taken by IBD patients only reflects what is recorded in clinical notes and does not incorporate external sources. The integration of a centralized medication data system into our IBD Data Lake is an ongoing process and is expected to be completed in the near future.

## Conclusion

We have successfully established and validated a novel IBD Data Lake that integrates machine learning and NLP. IBD patients were identified with high diagnostic accuracy using ICD-10 code queries, and the machine learning and NLP components allow for a comprehensive and timely extraction and organization of unstructured data. This will ultimately lay the groundwork for recruiting, following, and studying specific IBD cohorts of interest to address the gaps that remain in our knowledge. Exploring the emerging potential of LLMs and generative AI within the IBD Data Lake or similar data infrastructures could foster substantial innovation in the fields of IBD and gastroenterology.

### Key messages

What is already known?

- Despite substantial progress in advanced inflammatory bowel disease (IBD) therapies over the last decade, there remains a large proportion of patients who are non-responders or develop significant adverse events.

- Accurate data registries may help clinicians and researchers deepen their understanding of IBD and generate opportunities for personalized medicine to optimize overall patient outcomes. However, current data registries are often constrained by time and resources for patient data collection.

What is new?

**-** A novel inflammatory bowel disease repository (IBD Data Lake) that integrates machine learning and natural language processing has been established and validated.

**-** It demonstrates the ability to accurately identify IBD patients and facilitates comprehensive and timely extraction of structured and unstructured data.

How can this study help clinicians, researchers, and patients?

**-** This registry, powered by artificial intelligence, ultimately lays the groundwork for recruiting, following, and studying specific IBD cohorts of interest to address the gaps that remain in our knowledge.

**-** The IBD Data Lake has the potential to drive innovation in the field of IBD and gastroenterology.
